# Supplementary material for: Development and psychometric evaluation of the CO-PARTNER tool for collaboration and parent participation in neonatal care
Source: PLoS One. 2021 Jun 9;16(6):e0252074. doi: 10.1371/journal.pone.0252074 (PMC8189480; doi:10.1371/journal.pone.0252074)
Supplement: S1 File — (DOCX) [file pone.0252074.s001.docx]

[S1. The study on the effects of FICare (the AMICA study) 2](#_Toc72075736)

[**S1. Supplemental Figure 1.** Single Family Room in the new facility with possibility for parents to stay at all times 3](#_Toc72075737)

[S2. Language Considerations 4](#_Toc72075738)

[S3. Description of the neonatal population in the Netherlands 4](#_Toc72075739)

[S4. Other questionnaires used in the study for hypotheses testing. 5](#_Toc72075740)

[S5. Sample size calculation 7](#_Toc72075741)

[S6. Missing data model 8](#_Toc72075742)

[S7. Answers on statements of tool 9](#_Toc72075743)

[**S7. Supplemental Table 1.** Collaborative outcomes 9](#_Toc72075744)

[**S7. Supplemental Table 2.** Dichotomous measurement 10](#_Toc72075745)

[**S7. Supplemental Table 3.** Continuous measurement 11](#_Toc72075746)

[S8. Sensitivity analyses 12](#_Toc72075747)

[**S8. Supplemental Table 4.** Sensitivity analyses for missing data model and use of quartiles 12](#_Toc72075748)

[S9. Structural validity - Internal consistency 13](#_Toc72075749)

[**S9. Supplemental Table 5.** Composite Reliability 13](#_Toc72075750)

[S10. Construct validity - Distinctiveness 14](#_Toc72075751)

[**S10. Supplemental Table 6.** Average Variance Extracted 14](#_Toc72075752)

[**S10. Supplemental Table 7**. Distinctiveness of Domains 15](#_Toc72075753)

[S11. Construct validity - Hypothesis testing 16](#_Toc72075754)

[**S11. Supplemental Table 8.** Hypothesis testing with correlation results 16](#_Toc72075755)

[**S11. Supplemental Table 9.** Hypothesis testing, Regression results 19](#_Toc72075756)

[**S11. Supplemental Figure 2**. Differences in Domain 5 outcomes 20](#_Toc72075757)

[References to the supplement 21](#_Toc72075758)

# **S1. The study on the effects of FICare (the AMICA study)**

We used data during a multicentre non-randomised prospective study on the effects of FICare on infants and their parents in a NICU level 2 context in the Netherlands [1]. Below description is adapted from van Veenendaal et al. [2].

**Intervention**

*Family Integrated Care (FICare)*

In the FICare model parents were trained to be the primary caregiver of their infant, and nurses supported, instructed and counseled parents. Parents were invited but not obligated to be present 8 hours per day, and rooming-in facilities were present if they wanted to stay during the night. Parents were actively encouraged to participate in their infant’s care by providing feedings by nasogastric tube, breast or bottle, providing skin-to-skin care (by mothers and fathers), weighing and regulating temperature control.

Family-centered rounds were implemented and included active parental participation in medical decision making on daily medical rounds and involving them in the process of patient management together with the nurses and doctors [3,4]. Weekly, parents had group-sessions to learn and talk about prematurity and their infant’s hospital stay, guided by healthcare professionals or veteran parents [5].

Nurses provided cardiorespiratory monitoring as well as treatments such as intravenous fluids or antibiotics, placing nasogastric tubes, providing respiratory support and phototherapy [5–7].

*Single Family Rooms*

The new neonatal, maternity and obstetric ward of the hospital allowed mothers and their infants to always stay together in one family room. They were never separated, even when one or both needed medical care (providing complete couplet-care, Supplemental Figure S1) [8]. Other family members could also be present 24 hours per day. With this new architectural design of the mother-child-center, maternity and neonatology services were fully integrated, with trained professionals with special skills to provide simultaneous medical care for ill mothers and/or preterm infants.

**Control/reference treatment**

*Standard Neonatal Care in Open Bay Units*

Standard neonatal care in OBU was in the other facility of the hospital. In this ward infants stayed together in an open bay ward (a maximum of 18 infants in the ward), with incubators and beds lined up next to each other separated by curtains (Supplemental Figure S2 (online)). The OBU was close to, but physically separated from the maternity ward. Parents could visit their child, participate in routine infant care, and provide skin-to-skin contact. Due to the setting, the duration mothers and fathers could stay at the bedside of their infant was limited. Rooming-in facilities were not present.

During the hospital stay of their infant, parents were stimulated by the nurses to take part in the basic care of their infant. Medical rounds were done in a separate room from the OBU, attended by the nurses and the doctors, without the parents. Approximately two days before discharge, parents could room-in with their infant in a family room near the neonatal ward.

**Inclusion- and exclusion criteria**

Infants were included if they were born between 24 and 36 6/7 weeks’ gestation, with a post-conceptional age <44 weeks on admission and if they had a length of hospital stay in the level 2 ward of 3 days. Infants were excluded if they were born abroad (not in the Netherlands) or had a congenital anomaly (for example severe congenital heart-defects requiring surgery or Down’s syndrome).

## **S1. Supplemental Figure 1.** Single Family Room in the new facility with possibility for parents to stay at all times


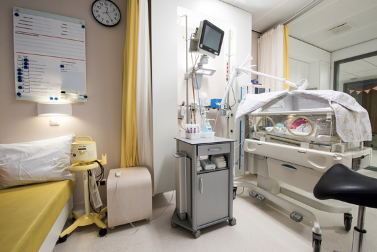


#

# **S2. Language Considerations**

The initial IPP and literature searches for definitions of parent participation were conducted primarily in English. The location of the pilot test within the larger intervention study was in Amsterdam, The Netherlands. The project leader is bilingual in English and Dutch. Forward translations of the 26 items from the IPP were completed from English to Dutch in duplicate. Forward translation occurred of both the parent participation definition and the 26 items from the IPP, and the following 62 items were obtained through Dutch language interviews. The 88 items were evaluated by Dutch speaking experts and parents. The final 34 items (CO-PARTNER) were used in a large Dutch language intervention study.

# **S3. Description of the neonatal population in the Netherlands**

Altered after van Veenendaal et al. [2].

In the Netherlands 17% of infants is born by cesarean section (8,1% primary and 7.9% secondary cesarean section) and approximately 70% of births occur in hospital (vs 30% in the community). In the Netherlands, 6.9% of infants are born preterm, and in the hospital region of Amsterdam the perinatal mortality rate (from 22 weeks of gestation up to 28 days after birth) is 0.56% (0.69% in the Netherlands) [9].

Different populations of preterm infants are defined within neonatal care in the Netherlands; Intensive care patients (IC), post-intensive high care patients, high-care (HC) patients and medium-care (MC) patients. *Intensive care infants*: Infants who need intensive care (e.g. cardiorespiratory support) are referred to a level 3 neonatal intensive care unit (NICU). In 2010, the Dutch guideline on perinatal practice in extremely premature delivery lowered the limit offering intensive care from 25+0 to 24+0 weeks of gestational age [10]. *Post-Intensive High Care infants*: Infants who are expected to need intensive care (gestational age <32 weeks and/or expected birth weight <1200 gram) are born in one of the ten level 3 NICUs. Once these preterm infants are stable and their actual weight is (around) 1000 gram or more, they are referred to a hospital with a (licensed) Post-Intensive-Care unit (level-2 Neonatal Ward). These Post-Intensive High Care infants often are still on non-invasive respiratory support, and/or have central venous catheters for parenteral nutrition or receive multiple medications. *High care infants*: Infants who are usually born in a level 2 neonatal ward, with a gestational age of at least 32 0/7 weeks and an expected birth weight >1200 grams. They also can be treated with non-invasive respiratory support, parenteral nutrition, central venous catheters and other medication. *Medium care infants*: Infants who are usually stable, growing infants without the need for cardiorespiratory monitoring or respiratory support, but can be treated with parenteral nutrition or medication by peripheral venous access. This group also consists of (near-) term small- or large for gestational age infants with glucose monitoring, stable infants treated with antibiotics for suspected perinatal infection, intravenous treatment of hypoglycemia or phototherapy for hyperbilirubinemia.

# **S4. Other questionnaires used in the study for hypotheses testing.**

**Hospital Anxiety and Depression Scale**

The Hospital Anxiety and Depression Scale (HADS) is developed in order to provide a screening measure for the presence of anxiety and depression. It contains two 7-item scales: one for anxiety and one for depression both with a score range of 0-21. It has been validated in Dutch before in ages 16 to 65 years [11].

**PMP-SE**

The Perceived (Maternal) Parenting Self-Efficacy (PMP-SE) tool, was used to measure perceived parental self-confidence when caring for the infant admitted to the Neonatal Ward [12].The internal consistency reliability of the Perceived Maternal Parenting Self-Efficacy tool is 0.91, external/test-retest reliability is 0.96. A total of four conceptually unique subscales of parenting are: “Care taking procedures” (parents’ perceptions of their ability to perform the activities and tasks related to the baby’s basic needs like feeding). “Evoking behaviour(s)” (perceptions in their ability to elicit a change in the baby’s behaviour, for example, soothing the baby). “Reading behaviour(s) or signalling” (perceptions in their ability to understand and identify changes in their baby’s behaviour, for example, ‘I can tell when my baby is sick’). “Situational beliefs”(parents’ beliefs about their ability to judge their overall interaction with the baby). Responses to each item were recorded on a four point Likert scale ranging from ‘strongly disagree’ (score 1) to ‘strongly agree’ (score 4). A low score on this scale indicates a low parental self-efficacy.

**EMPATHIC-N**

Parental satisfaction with participation was measured using the EMpowerment of PArents in THe Intensive Care - Neonatology questionnaire [13]. This questionnaire was developed and tested in a single center in the Netherlands, and available in Dutch. The domains covered are: Information (14 statements); Care and Treatment (20 statements); Parental Participation (nine statements); Organization (11 statements); and Professional Attitude (13 statements). The 57 statements divided in five domains provide a conceptualization of parent satisfaction within the neonatal ward from a family-centred care perspective. We used the parent participation items for this study.

**PBQ**

The Postpartum Bonding Questionnaire (PBQ) [14], was devised as a screening instrument to detect bonding problems in obstetric and primary care services. The PBQ is a 25- item scale reflecting a mother’s feelings or attitudes towards her baby (e.g. ‘‘I feel close to my baby’’, ‘‘My baby irritates me’’). Participants rate how often they agree with these statements on a 6-point Likert scale ranging from always (score=0) to never (score=5) with low scores denoting good bonding. The PBQ has four subscales which reflect impaired bonding (Scale 1) (12 items, ranging from 0 to 60), rejection and anger (Scale 2) (7 items, scores ranging from 0 to 35), anxiety about care (Scale 3) (4 items, scores ranging from 0 to 20) and risk of abuse (Scale 4) (2 items, scores ranging from 0 to 10). Scale 1 (impaired bonding) has a sensitivity of 0.93 and a specificity of 0.85 in detecting mothers with a bonding disorder

**PSS-NICU**

*The Parental Stressor Scale*:*Neonatal Intensive Care Unit*  is a scale to measure parental perceptions of stressors associated with the hospitalisation of their child [15]. It measures parents’ perceptions of stressors arising from the physical and emotional environment. It takes in account the infant’s behavior and appearance, parental role alterations, and the sights and sounds of the environment. The PSS:NICU has been translated into Dutch. It has a minimum score of 46, and a maximum score of 230. Previous internal consistencies have been 0.89 - 0.94.

#

# **S5. Sample size calculation**

For the AMICA study we performed a sample size calculation for the primary outcome of neurodevelopment in preterm infants at 2 years of age [23]. We pre-stratified the study population towards infants born <32 weeks of gestation with a previous admission to a level 3 NICU and infants that were born >32 weeks of gestation. Within each gestational age group, we did a power calculation for the primary outcome of neurodevelopment. We calculated to have 64 experimental subjects and 128 control subjects with power 0.90 (β) at a significance level of 0.05 (α) with a true difference in the outcome of neurodevelopment of 2 years of ½ SD. To allow for 30% withdrawal we aimed to include 91 patients in FICare and 182 patients in standard care per risk group (post-intensive care versus inborn infants). A total of 546 infants who were hospitalised, and their parents were expected to be included in this study.

# **S6. Missing data model**

The following auxiliary variables were used in the imputation model:

- Length of stay in hospital
- Gestational age
- Gemelli status / singleton
- Inborn in hospital (or referred from level 3 NICU)
- Mode of delivery
- Age of parent
- Father/mother
- Admitted to FICare or SNC setting
- Identified with Dutch Culture
- Working hours pre-pregnancy
- Experienced stress during pregnancy (scale 1-5)
- First child to raise
- Highest education
- Smoking
- Attendance to FICare meetings in hospital stay
- Support by child psychologist during hospital stay
- Parcel depression and anxiety score at discharge (HADS)
- Parcel satisfaction score at discharge (EMPATHIC-N)
- Parcel parcel self-efficacy score at discharge (PMP-SE)
- Parcel stress score at discharge (PSS-NICU)
- Parcel parent-infant bonding score at discharge (PBQ)

Not included in multiple imputed model due to multicollinearity:

- Time spent in hospital (level 2 and total hospital stay)
- Birthweight
- Working status
- Post-intensive care status
- Inborn in level 2 status
- FICare sessions during hospital stay
- Intention to raise child with partner

# **S7. Answers on statements of tool**

## **S7. Supplemental Table 1.** Collaborative outcomes

|  | Completed answer (n) | The nurse does this (n, (%)) | We do this together (n, (%)) | I do this independently (n, (%)) | This was not applicable (n, (%)) |
| --- | --- | --- | --- | --- | --- |
| Domain 1: Participation in  Daily Care |  |  |  |  |  |
| 1. Bath my child/clean my child with a washcloth. | 304 | 5 (1.65) | 90(29.61) | 180(59.21) | 29(9.54) |
| 2. Change my child’s diaper. | 303 | 0(0) | 8(2.64) | 292 (96.37) | 3(0.99) |
| 3. Feed my child (breast or bottle). | 303 | 1(0.33) | 30(9.90) | 265(87.46) | 7(2.31) |
| 4. Change my child’s clothing. | 304 | 1(0.33) | 14(4.61) | 284(93.42) | 5(1.65) |
| 5. Get my child out of the incubator/cradle. | 302 | 2(0.66) | 19(6.29) | 274(90.73) | 7(2.32) |
| 6. Give my child medication. | 302 | 114(37.75) | 42(13.91) | 89(29.47) | 57(18.87) |
| 7. Weigh my child. | 300 | 50(16.67) | 113(37.67) | 127(42.33) | 10(3.33) |
| 8a. Keep track of defecation of my child. | 299 | 57(19.60) | 134(44.82) | 95(31.77) | 13(4.35) |
| 8b. Keep track of urination. | 299 | 58(19.40) | 125(41.81) | 103(34.45) | 13(4.35) |
| 9. Measuring the temperature of my child. | 298 | 14(4.70) | 52(17.45) | 230(77.18) | 2(0.671) |
| 10. Keep track of my child’s weight. | 295 | 50(16.95) | 150(50.85) | 87(29.49) | 8(2.71) |
| 11. Keep track of drinking and my child’s feeds. | 292 | 41(14.04) | 143(48.97) | 105(35.96) | 3(1.03) |
| Domain 2: Participation in  Medical Care |  |  |  |  |  |
| 12. Giving tube feeding to my child. | 304 | 62(20.40) | 117(38.49) | 94(30.92) | 31(10.20) |
| 13. Looking at my child’s monitor, and handling accordingly (e.g. stimulating during a bradycardia). | 301 | 59(19.60) | 79(26.25) | 59(19.60) | 104(34.55) |
| 14. Regulating the visiting of others to my child. | 292 | 1(0.34) | 28(9.59) | 239(81.85) | 24(8.22) |
| 15. Participating in the daily rounds with the doctor. | 291 | 55(18.90) | 95(32.65) | 95(32.65) | 46(15.81) |
| Domain 6: Closeness and Comforting the Infant |  |  |  |  |  |
| 25. Holding/rock/cuddle my child. | 305 | 0(0) | 8(2.62) | 296(97.05) | 1(0.33) |
| 26. Comfort my child whenever he/she needs it. | 282 | 1(0.36) | 20(7.09) | 253(89.72) | 8(2.84) |
| 27. Kangaroo-mother care-skin to skin contact. | 296 | 0(0) | 16(5.41) | 268(90.54) | 12(4.05) |
| 28. Being together with my child, being close with my child. (intimate time). | 281 | 0(0) | 8(2.85) | 271(96.44) | 2(0.71) |
| 29. Being together with my child (being present). | 279 | 0(0) | 10(3.58) | 267(95.70) | 2(0.72) |
| 30. Soothe my child during a painful procedure (for instance drawing blood). | 285 | 14(4.91) | 68(23.86) | 167(58.60) | 36(12.63) |
| 31. Recognizing my child’s signals. | 291 | 3(1.03) | 146(50.17) | 136(46.74) | 6(2.06) |

##

| Removed Items | Completed answer (n) | The nurse does this (n, (%)) | We do this together (n, (%)) | I do this independently (n, (%)) | This was not applicable (n, (%)) |
| --- | --- | --- | --- | --- | --- |
| Walking a small round with my child if it is permitted. | 280 | 0(0) | 3(1.07) | 135(48.21) | 142(50.71) |

## **S7. Supplemental Table 2.** Dichotomous measurement

|  | Completed answer (n) | Yes (n, (%)) | (No (n, (%)) |
| --- | --- | --- | --- |
| Domain 3: Acquiring Information |  |  |  |
| 16. Did you ask health care professionals information on the health of your child? | 301 | 287(95.35) | 14(4.65) |
| 17. Did you ask the healthcare professionals for information about your child for times when you were not present? | 301 | 274(91.03) | 27(8.97) |
| 18. Did you talk with another parent about your experiences? | 302 | 190(62.91) | 112(37.09) |
| Domain 4: Parent Advocacy |  |  |  |
| 19. I stuck up for my child; I told somebody to do something in the care of my child. | 301 | 240(79.73) | 61(20.27) |
| 20. I stuck up for my child; I told somebody NOT to do something in the care of my child; I gave boundaries | 301 | 174(57.81) | 127(42.19) |
| 21. I gave an explanation on the daily routines of my child to a healthcare professional. | 301 | 153(50.83) | 148(49.17) |

## **S7. Supplemental Table 3.** Continuous measurement

|  | Completed answer (n) | Mean (SD) |
| --- | --- | --- |
| Domain 5: Time Spent with Infant |  |  |
| 22. On average, how many hours per day were you present in the hospital with your child? | 303 | 11.58 (8.12) |
| 23. How many hours per day do you have contact with your child? | 301 | 6.84(5.30) |
| 24. How many hours per day were you really close with your child? | 294 | 4.33(3.58) |

#

| Removed Items  Continuous Variable | Completed answer (n) | Mean (SD) |
| --- | --- | --- |
| If ‘Kangaroo-mother care-skin to skin contact’ is not equal to ‘This is not applicable’ answer this question: Please indicate how much kangaroo mother care/skin to skin contact you had on average during hospital stay **(How many times per day)** | 276 | 12.36 (2.25) |
| If ‘Kangaroo-mother care-skin to skin contact’ is not equal to ‘This is not applicable’ answer this question: Please indicate how much kangaroo mother care/skin to skin contact you had on average during hospital stay **(How many minutes per skin-to-skin session)** | 201 | 24.34(5.82) |

# **S8. Sensitivity analyses**

## **S8. Supplemental Table 4.** Sensitivity analyses for missing data model and use of quartiles

| Fit parameter | Estimate (-1 DB) | Estimate (-1 DB)  QUARTILES database | Estimate (NA DB) | Estimate (NA DB)  QUARTILES database |
| --- | --- | --- | --- | --- |
| RMSEA | 0.043 (0.037; 0.049) | 0.045 (0.040; 0.051) | 0.008 (0.000; 0.021) | 0.000 |
| CFI (scaled) | 0.846 (0.692) | 0.816 (0.665) | 1.000 (0.554) | 1.000 (0.865) |
| TLI (scaled) | 0.834 (0.667) | 0.801 (0.638) | 1.264 (0.518) | 1.448 (0.854) |
| FMI (scaled) | 0.169 (0.059) | 0.133 (0.052) | 0.444 (0.221) | 0.406 (0.212 |
| SRMR | 0.128 | 0.129 | 0.153 | 0.155 |

NA DB: missing database

#

# **S9. Structural validity - Internal consistency**

## **S9. Supplemental Table 5.** Composite Reliability

| Domain | Composite Reliability Score |
| --- | --- |
| Daily care (Domain 1) | 0.934 |
| Medical care (Domain 2) | 0.558 |
| Acquiring Information (Domain 3) | 0.745 |
| Parent Advocacy (Domain 4) | 0.855 |
| Time spent with infant (Domain 5) | 0.839 |
| Closeness and comforting the infant (Domain 6) | 0.871 |

#

# **S10. Construct validity - Distinctiveness**

## **S10. Supplemental Table 6.** Average Variance Extracted

| Latent Variable | Average Variance Extracted (AVE) |
| --- | --- |
| Daily care (Domain 1) | 0.859 |
| Medical care (Domain 2) | 0.696 |
| Acquiring Information (Domain 3) | 0.833 |
| Parent Advocacy (Domain 4) | 0.900 |
| Time spent with infant (Domain 5) | 0.952 |
| Closeness and comforting the infant (Domain 6) | 0.822 |

#

## **S10. Supplemental Table 7**. Distinctiveness of Domains

| Latent Variable | Daily Care (Domain 1) | Medical Care  (Domain 2) | Acquiring Information  (Domain 3) | Parent Advocacy  (Domain 4) | Time Spent with Infant  (Domain 5) | Closeness  And Comforting  the Infant  (Domain 6) |
| --- | --- | --- | --- | --- | --- | --- |
| Daily Care  (Domain 1) | - |  |  |  |  |  |
| Medical Care  (Domain 2) | 0.768 | - |  |  |  |  |
| Acquiring Information  (Domain 3) | 0.270 | 0.460 | - |  |  |  |
| Parent Advocacy  (Domain 4) | 0.380 | 0.514 | 0.379 | - |  |  |
| Time Spent  with Infant  (Domain 5) | 0.347 | 0.219 | 0.199 | 0.409 | - |  |
| Closeness and Comforting  the Infant  (Domain 6) | 0.753 | 0.711 | 0.329 | 0.263 | 0.307 | - |

*(square root of AVE should be higher than latent variable correlation)

Heterotrait-Monotrait ratio criterion [16].

# **S11. Construct validity - Hypothesis testing**

## **S11. Supplemental Table 8.** Hypothesis testing with correlation results

| Outcomes |  | Pearson (r) | 95%CI | P-value |
| --- | --- | --- | --- | --- |
| Depression (HADS) | CO-PARTNER Total | -0.141 | -0.240 ; -0.029 | 0.0141 |
|  | Domain 1 | -0.178 | -0.285 ; -0.066 | 0.0020 |
|  | Domain 2 | -0.008 | -0.120 ; 0.105 | 0.892 |
|  | Domain 3 | -0.021 | -0.135 ; 0.093 | 0.714 |
|  | Domain 4 | -0.002 | -0.114 ; 0.111 | 0.977 |
|  | Domain 5 | -0.056 | -0.168 ; 0.058 | 0.336 |
|  | Domain 6 | -0.151 | -0.261 ; -0.038 | 0.009 |
| Self-efficacy (PMP-SE) | CO-PARTNER Total | +0.228 | +0.117 ; +0.332 | <0.0001 |
|  | Domain 1 | +0.185 | +0.073 ; 0.292 | 0.012 |
|  | Domain 2 | +0.124 | +0.011 ; +0.236 | 0.032 |
|  | Domain 3 | +0.033 | -0.080 ; +0.146 | 0.568 |
|  | Domain 4 | +0.095 | -0.021 ; +0.208 | 0.109 |
|  | Domain 5 | +0.164 | +0.031; +0.273 | 0.005 |
|  | Domain 6 | +0.181 | +0.067 ; +0.289 | 0.002 |
| Satisfaction (EMPATHIC-N) | CO-PARTNER Total | +0.197 | +0.080 ; +0.308 | 0.001 |
|  | Domain 1 | +0.147 | +0.032 ; +0.259 | 0.012 |
|  | Domain 2 | +0.105 | -0.009 ; +0.215 | 0.071 |
|  | Domain 3 | +0.111 | -0.016; +0.235 | 0.087 |
|  | Domain 4 | +0.020 | -0.104 ; 0.143 | 0.758 |
|  | Domain 5 | +0.154 | +0.039 ; +0.266 | 0.009 |
|  | Domain 6 | +0.165 | +0.041 ; +0.284 | 0.009 |
| Parent-infant bonding* (PBQ) | CO-PARTNER Total | -0.196 | -0.302 ; -0.056 | <0.001 |
|  | Domain 1 | -0.185 | -0.293 ; -0.071 | 0.002 |
|  | Domain 2 | +0.002 | -0.112 ; +0.116 | 0.972 |
|  | Domain 3 | -0.043 | -0.162 ; +0.078 | 0.487 |
|  | Domain 4 | -0.063 | -0.177 ; +0.052 | 0.280 |
|  | Domain 5 | -0.166 | -0.274 ; -0.054 | 0.004 |
|  | Domain 6 | -0.169 | -0.278 ; -0.056 | 0.004 |
| Parent NICU stress (PSS-NICU) | CO-PARTNER Total | -0.056 | -0.169 ; +0.058 | 0.332 |
|  | Domain 1 | -0.060 | -0.172; +0.053 | 0.298 |
|  | Domain 2 | -0.074 | -0.185 ; +0.039 | 0.201 |
|  | Domain 3 | +0.131 | +0.018 ; +0.240 | 0.022 |
|  | Domain 4 | +0.003 | -0.110 ; +0.116 | 0.956 |
|  | Domain 5 | -0.040 | -0.153 ; 0.075 | 0.496 |
|  | Domain 6 | -0.044 | -0.159 ; 0.073 | 0.461 |

* Higher scores indicate *lower* bonding, and more parent-infant-bonding difficulties.

##

## **S11. Supplemental Table 9.** Hypothesis testing, Regression results

| Assumption | Outcome | Beta | 95%CI | P-value |
| --- | --- | --- | --- | --- |
| FICare versus Standard care | CO-PARTNER Total | +6.020 | +4.144 ; +7.895 | <0.0001 |
|  | Domain 1 | +1.051 | +0.591 ; +2.432 | 0.00138 |
|  | Domain 2 | +1.371 | +0.982 ; +1.760 | <0.0001 |
|  | Domain 3 | -0.216 | -0.372 ; -0.059 | 0.007 |
|  | Domain 4 | +0.745 | +0.513 ; 0.977 | <0.0001 |
|  | Domain 5 | +1.853 | +1.196 ; 2.509 | <0.0001 |
|  | Domain 6 | +0.616 | +0.228 ; +1.004 | 0.0020 |
| Mothers versus fathers | CO-PARTNER Total | +2.103 | +0.084 ; +4.121 | 0.041 |
|  | Domain 1 | +0.472 | -0.471 ; +1.415 | 0.326 |
|  | Domain 2 | -0.522 | -0.942 ; -0.103 | 0.015 |
|  | Domain 3 | -0.062 | -0.221 ; +0.098 | 0.445 |
|  | Domain 4 | +0.176 | -0.073 ; +0.425 | 0.164 |
|  | Domain 5 | +2.496 | +1.859 ; +3.132 | <0.0001 |
|  | Domain 6 | +0.007 | -0.0391 ; +0.406 | 0.971 |
| More present (Domain 5) | More daily care (Domain 1) | +0.390 | +0.240 ; +0.540 | <0.0001 |

FICare: family integrated care. 95%CI: 95% confidence interval

## **S11. Supplemental Figure 2**. Differences in Domain 5 outcomes


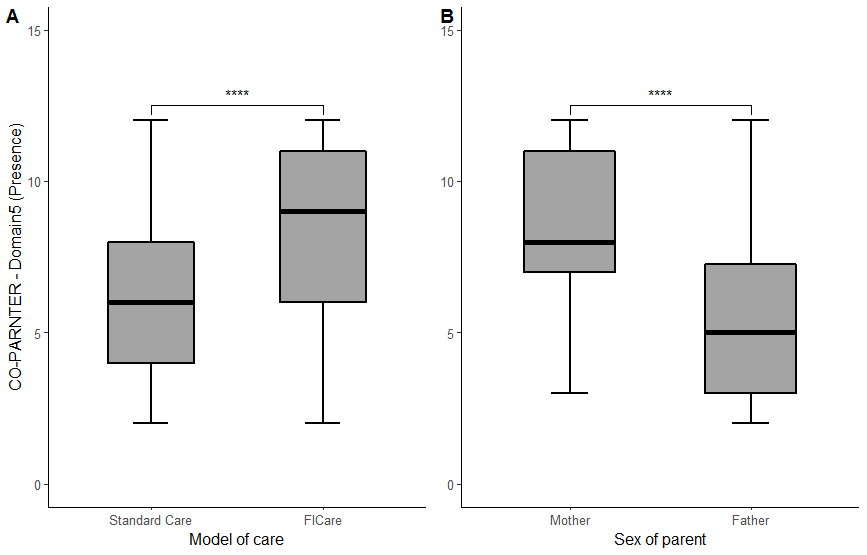


# **References to the supplement**

1. van Veenendaal NR, van Kempen AAMW, Maingay F, Recourt-Vollebregt M, van der Schoor SRD, van Goudoever J. Family Integrated Care in the Neonatal Ward - the AMICA study. 2017 [cited 19 Mar 2020]. Available: https://www.trialregister.nl/trial/6175

2. van Veenendaal NR, van der Schoor SRD, Heideman WH, Rijnhart JJM, Heymans MW, Twisk JWR, et al. Family integrated care in single family rooms for preterm infants and late-onset sepsis: a retrospective study and mediation analysis. Pediatr Res. 2020. doi:10.1038/s41390-020-0875-9

3. Davidson JE, Aslakson RA, Long AC, Puntillo KA, Kross EK, Hart J, et al. Guidelines for Family-Centered Care in the Neonatal, Pediatric, and Adult ICU. Crit Care Med. 2017;45: 103–128. doi:10.1097/CCM.0000000000002169

4. Voos KC, Ross G, Ward MJ, Yohay AL, Osorio SN, Perlman JM. Effects of implementing family-centered rounds (FCRs) in a neonatal intensive care unit (NICU). J Matern Neonatal Med. 2011;24: 1–4. doi:10.3109/14767058.2011.596960

5. O’Brien K, Robson K, Bracht M, Cruz M, Lui K, Alvaro R, et al. Effectiveness of Family Integrated Care in neonatal intensive care units on infant and parent outcomes: a multicentre, multinational, cluster-randomised controlled trial. Lancet Child Adolesc Heal. 2018;2: 245–254. doi:10.1016/S2352-4642(18)30039-7

6. Galarza-Winton ME, Dicky T, O’Leary L, Lee SK, O’Brien K. Implementing family-integrated care in the NICU: Educating nurses. Adv Neonatal Care. 2013;13: 335–340. doi:10.1097/ANC.0b013e3182a14cde

7. Jiang S, Warre R, Qiu X, O’Brien K, Lee SK. Parents as practitioners in preterm care. Early Hum Dev. 2014;90: 781–785. doi:10.1016/j.earlhumdev.2014.08.019

8. Stelwagen MA, van Kempen AAMW, Westmaas A, Blees YJ, Scheele F. Integration of maternity and neonatal care to empower parents. JOGNN - J Obstet Gynecol Neonatal Nurs. 2020;49: 65–77. doi:10.1016/j.jogn.2019.11.003

9. Perined, van Dijk A, Dijs-Elsinga J, de Miranda E, Blaauw J, Klumper F, et al. Perined. Perinatale Zorg in Nederland 2016. Utrecht; 2016.

10. de Laat MWM, Wiegerinck MM, Walther FJ, Boluyt N, Mol BWJ, van der Post JAM, et al. Richtlijn “Perinataal beleid bij extreme vroeggeboorte”. Ned Tijdschr Geneeskd. 2010;154.

11. Spinhoven P, Ormel J, Sloekers PPA, Kempen GIJM, Speckens AEM, Van Hemert AM. A validation study of the Hospital Anxiety and Depression Scale (HADS) in di?erent groups of Dutch subjects. Psychol Med. 1997. doi:10.1016/S0163-8343(03)00043-4

12. Barnes CR, Adamson-Macedo EN. Perceived Maternal Parenting Self-Efficacy (PMP S-E) tool: Development and validation with mothers of hospitalized preterm neonates. J Adv Nurs. 2007;60: 550–560. doi:10.1111/j.1365-2648.2007.04445.x

13. Latour JM, Duivenvoorden HJ, Hazelzet JA, Van Goudoever JB. Development and validation of a neonatal intensive care parent satisfaction instrument. Pediatr Crit Care Med. 2012;13: 554–559. doi:10.1097/PCC.0b013e318238b80a

14. Brockington IF, Fraser C, Wilson D. The Postpartum Bonding Questionnaire: A validation. Arch Womens Ment Health. 2006;9: 233–242. doi:10.1007/s00737-006-0132-1

15. Miles MS, Funk S, Carlson J. Parental Stressor Scale: Neonatal Intensive Care Unit. Nurs Res. 1993;42: 148–152.

16. Ab Hamid MR, Sami W, Mohmad Sidek MH. Discriminant validity assessment: use of Fornell & Larcker criterion versus HTMT criterion. Journal of Physics: Conference Series. 2017. doi:10.1088/1742-6596/890/1/012163
