# Supplementary material for: Development and psychometric evaluation of the CO-PARTNER tool for collaboration and parent participation in neonatal care
Source: PLoS One. 2021 Jun 9;16(6):e0252074. doi: 10.1371/journal.pone.0252074 (PMC8189480; doi:10.1371/journal.pone.0252074)
Supplement: Attachment — Submitted filename: renamed_bfe4b.docx [file pone.0252074.s002.docx]

**COMMENTS – Manuscript PONE-D-20-36004**

Title: “Development and psychometric evaluation of the CO-PARTNER tool for collaboration and parent participation in neonatal care”

**Important note: This review pertains only to ‘statistical aspects’ of the study and so** **‘****clinical aspects’ [like medical importance,** **relevance of the study,** **‘clinical significance and** **implication(s)’ of the whole study, etc.] are to be evaluated [should be assessed] separately/independently. Further please note that any ‘statistical review’ is generally done under the assumption that (such) study specific methodological [as well as execution] issues are perfectly taken care of by the investigator(s). This review is not an exception to that and so does not cover clinical aspects {however, seldom comments are made only if those issues are intimately / scientifically related & intermingle with ‘statistical aspects’ of the study}. Agreed that ‘statistical methods’ are used as just tools here, however, they are vital part of methodology [and so should be given due importance].**

**COMMENTS:** Actually this excellent study which developed and psychometrically evaluated a tool measuring active parent participation and collaboration in neonatal care is most welcome, however, I wonder to note that the refer number 40 {one which is otherwise important} is quoted only in the context of ‘values for CR are between 0.6 and 0.9 [(40)](https://www.zotero.org/google-docs/?kovtfE)’ in line 198. Reference 40 [‘Discriminant Validity Assessment: Use of Fornell & Larcker criterion versus HTMT Criterion’, M. Hamid, Waqas Sami, M. Sidek, Published 2017 with DOI:10.1088/1742-6596/890/1/012163] compares Fornell and Larcker criterion with Heterotrait-Monotrait (HTMT) ratio of correlations which is a new method emerged for establishing the discriminant validity assessment.

I fully agree that the Fronell-Larcker criterion is one of the most popular techniques used to check the discriminant validity of measurements models {though I am not really very familiar/no-first-hand-experience with these techniques [which probably is the reason of raising such question(s)]}. According to this criterion, the convergent validity of the measurement model can be assessed by the Average Variance Extracted (AVE) and Composite Reliability (CR).

However, recent research suggests that the Fornell-Larcker criterion is not effective under certain circumstances pointing to a potential weakness in the most commonly used discriminant validity criterion (??). Assessment of discriminant validity is a must in any research that involves latent variables for the prevention of multicollinearity issues. It is well-known that Fornell and Larcker criterion is the most widely used method for this purpose. However, a new method should have been discussed/mentioned, I guess.

Use of COSMIN checklist for assessing the methodological quality is most appreciated (linen 90). Why you say “for this substudy”, whereas, this is a full study. Or have you used COSMIN checklist only for some substudy/subsample? As pointed out in reference 32, it is true that ‘Missing data in a multi-item instrument were best handled by multiple imputation at the item score level’.

What is ‘Singleton’ [n=260, (72.6 %)] in Table 2 on ‘Baseline characteristics of the sample’. Just out of curiosity – Do they differ from others/remining?

CONGRATULATIONS on rigorous instrument development and psychometric testing methodology to develop a measure of parent participation and partnership in neonatal care.
