# Supplementary material for: Development and psychometric evaluation of the CO-PARTNER tool for collaboration and parent participation in neonatal care
Source: PLoS One. 2021 Jun 9;16(6):e0252074. doi: 10.1371/journal.pone.0252074 (PMC8189480; doi:10.1371/journal.pone.0252074)
Supplement: Attachment — Submitted filename: Reviewer comments Dec 2020 Copartner.docx [file pone.0252074.s003.docx]

**Review on paper titled: Development and psychometric evaluation of the CO-PARTNER tool for collaboration and parent participation in neonatal care**

**Comments to authors:**

This manuscript adds valuable information on the assessment of the active participation of parents within the NICU. The manuscript clearly explains the process of item generation and the participatory method used is appreciated. The possible application of the new tool in the field is exciting! However, there are several instances where some clarification could assist the reader in understanding the flow of the study and the results. The many references to the supplemental information make this manuscript challenging to follow and incorporating more of the information through brief descriptions of the content of the tables may be more beneficial than simply referring to the tables. Maybe consider dividing the manuscript into 2 papers, one focusing on the development of the tool and the item generation and a second paper describing the construct validity, etc. It will be useful to clarify the hypotheses and the correlation with the results using consistent terminology. Please consider elaborating on the nature of the items included in the tool and highlight how it is related to collaboration as the manuscript makes several references to this construct however it is unclear how the tool explicitly assesses it. Limitations: please describe the limitations related to this study in addition to the limitations of the tool itself. Please clarify how the active independent or assisted completion of tasks are related to collaboration. There are several instances where participation and collaboration are used interchangeably, yet it is unclear for the reader how the items in the tool specifically assess collaboration as opposed to parental participation in caregiving and medical tasks. Please look through the reference list again as there were some inconsistencies with regard to capitalization.

**Minor concerns:**

Line 112: “22 parents participated in the focus groups to assist with item generation”

- Please include a rationale for selecting 22 parents. How were they recruited?

Line 132: “current state in the literature”(2)

- This statement would be better supported by a different article. The article cited may not be sufficient to conclude on the “current state of the literature” in a broad sense. Please consider rephrasing or supplementing the statement with additional citations.

Line 227-9: “For domain 3 and 4 ‘yes’ was scored as 1, and ‘no’ as 0 (minimum scores 0 to 3). Non-applicable items were transformed to 0 (no participation in this item).”

- Please clarify why 0 is used for both No and Not applicable?

In line 258 it states: “Factor loadings for domains are described in Table 3. Sensitivity analyses for missing data, revealed that model fit was better without transforming the non-applicable items (scored -1) to missing (see S7. Appendix).”

- This seems somewhat contradicting to the information stated in line 227-229?

Line 241: Please consider adding Postmenstrual age at time of assessment along with GA

Line 277: Table 3: Please consider repeating the header row for tables broken across two pages

References: Some inconsistency with the capitalization of titles noted throughout reference list

**Major concerns:**

Line 127: “a total of 34 items were generated.”

- This section leads directly into “Domains” and then table 1 where only 31 items appear. It may be useful to include the rationale for dropping items prior to Table 1 or refer the reader to the specific section where it is explained as the reader would expect to see 34 items in Table 1 based on the information provided in line 127. The rationale is only explained in line 250.

Line 247: Table 2

- Please explain why the number of parents included is lower than the number of families included
- Table 2 contains 2 lines for Gestational age and singletons: please explain why there is a difference between the first line and the second line under included infants. Please clarify what the top section of table 2 represents – is this the characteristics across the larger FICARE study?

Line 328: “The findings from this study should be considered in light of its limitations.” This section should deal with the limitations of this study yet it only lists the limitations of the tool. The tool limitations are important to mention, but study limitations should also be included. This also lends toward separation into two manuscripts: one on development followed by one on evaluating the tool.

Line 347: “This tool can be used for benchmarking across settings” This is a strong statement-consider softening.

Line 349: “With these scores parents can give feedback on how well they partnered with providers during care and **to what extent participation and collaboration** were possible for them during the hospital stay.”

- This section seems somewhat confusing. How will the parents be able to describe “ to what extent they could collaborate” – the tool only provides measures of whether the parents did tasks independently or not and does not ask a question related to the amount of collaboration in the unit.
- Some units across the globe rely on families to deliver care (out of necessity) and therefore completing a task independently is not necessarily a direct measure of collaboration between healthcare workers and parents – it is just a measure of parental active participation
- Domain 3 has dichotomous questions on whether parents requested information: If information was freely volunteered by health care staff, parents may not have needed to request it, yet it would not be a marker of decreased collaboration or participation.

Line 351: “Equally, the CO-PARTNER tool enables comparison of parent partner care practices and to study (health) outcomes in infants and their parents through, for instance, mediation analysis (51).”

- This statement does not seem pertinent to the current manuscript as infant outcomes were not discussed or reported.

Line 369:

“Likewise, the COPARTNER tool could be studied to evaluate progress within parents (beginning and end of hospital stay) or to **evaluate the effect of education program**s for parents and healthcare professionals on parent participation and collaboration.”-consider rewording to …”to evaluate changes in parent participation…”

Line 211: Hypothesis testing

- It may be useful to include the hypotheses in a previous section of the paper (introduction?) rather than in the statistics section. Please consider numbering the hypotheses for ease of referring back to them and linking it directly to the results obtained using consistent terminology.

| Hypotheses: line 211-223 | Results reported: line 280-293 | Comments |
| --- | --- | --- |
| “ A priori, we hypothesized that the total score would have a negative correlation with parent well-being outcomes such as depression and anxiety, of -0.3 to -0.5, meaning that if parents were depressed or anxious they would demonstrate lower active parent participation.” | “Negative correlations were present between total and domain scores on the CO-PARTNER tool with depression and impaired parent-infant bonding.”  “No correlations were found between the CO-PARTNER tool and parent NICU stress (total and domain scores).” | Line 282: “**Overall** the direction of correlation between the participation score met our prespecified  283 hypotheses (Fig 2 and S10. Appendix)”  The word “**overall”** seems misleading as no correlation between CO-Partner and NICU stress was found |
| “For self-efficacy and satisfaction we defined a positive correlation with our parent participation tool of +0.3 to +0.5.” | positive correlations for total and domain scores between parent participation and parent self-efficacy and parent empowerment. | The hypothesis mentions parental satisfaction, yet the result only refers to participation and self-efficacy – not parental satisfaction |
| “We hypothesized that the COPARTNER-tool would be able to discriminate between high and low parent presence and participation within the trial on the effect of FICare in SFR on parent and infant outcomes (19).” | “Parents in the FICare group had significantly higher CO-PARTNER total scores (beta 6.020, 95%CI 4.144; 7.895, p<0.0001) and higher subdomain scores than parents in the standard care group, except for domain 3 (*Acquiring Information*, see S10. Appendix).” | Please clarify if higher total scores are synonymous with presence? Explicitly state if this hypothesis was correct?  Infant outcomes were not reported in this manuscript. Please add some clarification on why this hypothesis was used for this specific study or clarify whether it related more to the overarching study |
| “Also we hypothesized that mothers would be more present (Domain 5) than fathers, as fathers in the Netherlands had on average 2-5 days of paternity leave, and resume to work quickly after birth during conduct of the study (42).” | “Likewise, mothers had higher COPARTNER scores than fathers (beta 2.103, 95%CI 0.084; 4.121, p=0.041).” |  |
| “We also hypothesized that parents who were more present (domain 5), would perform more daily tasks independently (domain 1).” | “Overall, parents who were present more (*Domain 5)* participated more in daily care (*Domain 1*, beta 0.390, 95%CI +0.240; +0.540, p<0.0001).” | Does participation in daily care mean the same as independent participation – the scale gave the option that the parent does a task with the nurse- this may mean participation, but not *independent* participation as hypothesized. |
